# Vacuolar Na^+^/H^+^ NHX-Type Antiporters Are Required for Cellular K^+^ Homeostasis, Microtubule Organization and Directional Root Growth

**DOI:** 10.3390/plants3030409

**Published:** 2014-08-29

**Authors:** Tyler McCubbin, Elias Bassil, Shiqi Zhang, Eduardo Blumwald

**Affiliations:** Department of Plant Sciences, University of California, Davis, CA 95616, USA; E-Mails: tjmccubbin@ucdavis.edu (T.M.); esbassil@ucdavis.edu (E.B.); shqzhang@ucdavis.edu (S.Z.)

**Keywords:** vacuole, potassium, salt, homeostasis, pH, root, growth, microtubule, cytoskeleton, curling, skewing, NHX

## Abstract

Na^+^/H^+^ antiporters (NHXs) are integral membrane transporters that catalyze the electroneutral exchange of K^+^ or Na^+^ for H^+^ and are implicated in cell expansion, development, pH and ion homeostasis and salt tolerance. *Arabidopsis* contains four vacuolar NHX isoforms (NHX1–NHX4), but only the functional roles for NHX1 and NHX2 have been assessed thus far. Colocalization studies indicated that NHX3 and NHX4 colocalize to the tonoplast. To investigate the role of all vacuolar NHX isoforms, a quadruple knockout *nhx1nhx2nhx3nhx4*, lacking all vacuolar NHXs, was generated. Seedlings of *nhx1nhx2nhx3nhx4* displayed significantly reduced growth, with markedly shorter hypocotyls. Under high K^+^, but not Na^+^, pronounced root skewing occurred in *nhx1nhx2nhx3nhx4*, suggesting that the organization of the cytoskeleton might be perturbed. Whole mount immunolabeling of cortical microtubules indicated that high K^+^ caused significant microtubule reorganization in *nhx1nhx2nhx3nhx4* root cells of the elongation zone. Using microtubule stabilizing (Taxol) and destabilizing (propyzamide) drugs, we found that the effect of K^+^ on *nhx1nhx2nhx3nhx4* root growth was antagonistic to that of Taxol, whereas elevated K^+^ exacerbated the endogenous effect of propyzamide on root skewing. Collectively, our results suggest that altered K^+^ homeostasis leads to an increase in the dynamics of cortical microtubule reorganization in *nhx1nhx2nhx3nhx4* root epidermal cells of the elongation zone.

## 1. Introduction

Ion homeostasis and pH regulation are essential cellular processes necessary for plant growth, development and responses to environmental stress. Na^+^/H^+^ antiporters (NHXs) are integral membrane proteins localized to the plasma membrane and endomembrane compartments that facilitate the electroneutral exchange of Na^+^ or K^+^ for H^+^, thereby maintaining both luminal pH, as well as cation homeostasis. The *Arabidopsis* genome encodes eight NHX homologs that have been grouped based on sequence similarity and function into three distinct classes: plasma membrane (NHX7/SOS1 and NHX8), endosomal/vesicular (NHX5, NHX6), as well as four that are vacuolar (NHX1, NHX2, NHX3, NHX4) [1,2]. We previously reported the vacuolar localization and function of two of the vacuolar NHX isoforms, NHX1 and NHX2 [3,4], that bear strong sequence homology to the putatively vacuolar NHX3 and NHX4. Other work has shown colocalization of AtNHX3 with vacuolar proteins δ-TIP and γ-TIP, as well as SYP22 in tobacco protoplasts, suggesting a vacuolar and PVC localization of AtNHX3 [5]. Similarly, constitutive expression of AtNHX3 in sugar beet (*Beta vulgaris* L.) has been shown to confer increased salt tolerance, but also has led to high K^+^ accumulation in roots when grown under high salt [6]. In addition, Liu *et al.* found that *Arabidopsis nhx3* null mutants were hypersensitive to K^+^ deficiency, further implicating NHX3 in K^+^ homeostasis [5]. AtNHX4 localization was suggested to be similar to NHX1 at the tonoplast [7], but additional experiments are required to demonstrate localization convincingly. Given that little is known about the roles of NHX3 and NHX4 in vacuolar ion and pH homeostasis, further investigations are warranted.

Potassium transport into the vacuole has been shown to be a key regulator of cell expansion [8]. Both NHX1 and NHX2 are necessary for the accumulation of vacuolar K^+^ and vacuolar alkalization, because the double knockout *nhx1nhx2* accumulated 70% less vacuolar K^+^ and had significantly more acidic vacuoles than comparable wild-type (WT) plants [3]. *nhx1nhx2* plants also exhibited reduced overall growth, had smaller cells in all tissues examined, as well as aberrant flower development, such as incomplete filament elongation and anther dehiscence, which resulted in significantly reduced seed set. Others studies also showed that *nhx1nh2* double knockouts have high cytosolic K^+^ [9], altered vacuole morphology, compromised turgor, severely impaired stomatal conductance and less daytime transpiration due to the lack of K^+^ accumulation in guard cell vacuoles [10]. One intriguing phenotype of *nhx1nhx2* was their high sensitivity to additional K^+^ in the growth media, with a severe reduction in overall growth [3]. This phenotype was not evident when *nhx1nhx2* plants were grown in the presence of equimolar concentrations of Na^+^ ions. The presence of high K^+^ also led to profound curling of seedling roots, suggesting that altered K^+^ homeostasis, due to the lack of NHX1 and NHX2 antiporters, altered directional root growth [3].

Root growth patterning is controlled by microtubule and cytoskeleton reorganization [11]. Cellular Na^+^ homeostasis can affect cytoskeleton-mediated root growth, and the effects of salinity on directional root growth have already been reported [12,13]. Little is known about the role(s) of cellular K^+^ homeostasis in microtubule organization. Here, we show that vacuolar NHX1 to NHX4 reside at the tonoplast. Using the quadruple knockout *nhx1nhx2nhx3nhx4*, lacking all four vacuolar NHXs, we show that vacuolar NHXs impart essential functions in K^+^ homeostasis that affect directional root growth and cytoskeletal dynamics. The quadruple knockout exhibits more pronounced phenotypes compared to the *nhx1nhx2* double knockout, including increased sensitivity to K^+^, suggesting that NHX3 and NHX4 have additional roles in cell expansion and K^+^ homeostasis.

## 2. Results and Discussion

**Figure A1:**
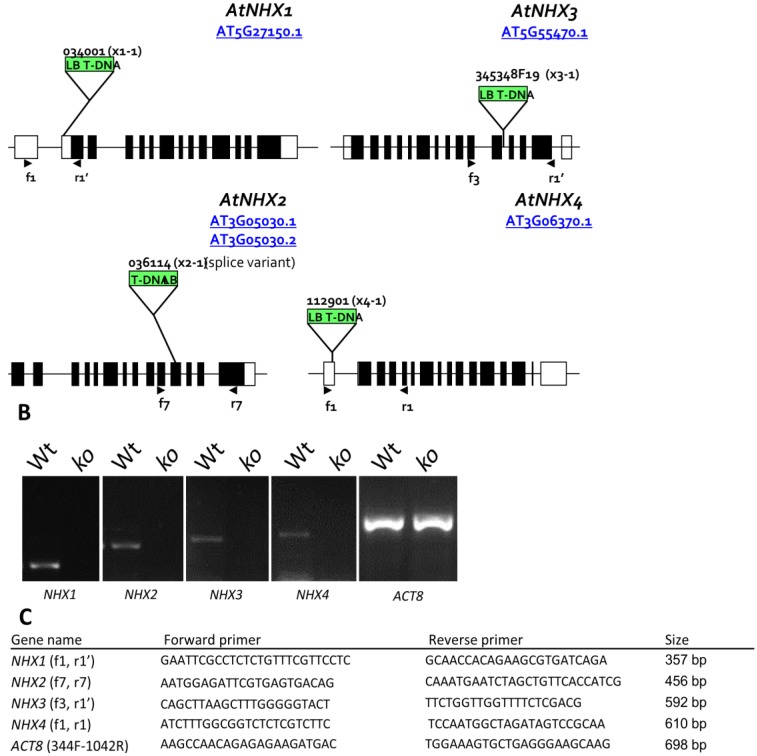
Genetic maps of *NHX* T-DNA insertions used in generating *nhx1nhx2nhx3nhx4* (**A**); rt-PCR shows no expression of *NHX1*, *NHX2*, *NHX3*, *NHX4* in *nhx1nhx2nhx3nhx4* (**B**); primers used for verifying gene expression are shown in the table (**C**).

We sought to assess the contribution of all vacuolar NHXs to overall plant growth and development by generating a quadruple knockout lacking all vacuolar NHX antiporters, NHX1, NHX2, NHX3 and NHX4. Using available T-DNA insertion lines (Figure A1), we generated the quadruple knockout *nhx1nhx2nhx3nhx4* through successive crosses of single, double and triple knockouts, as described in the Methods. The level of expression of all four vacuolar NHX transcripts in the *nhx1nhx2nhx3nhx4* knockout indicated that no full-length transcripts exist (Figure A1).

### 2.1. NHX3 and NHX4 Are Colocalized at the Tonoplast

**Figure 1:**
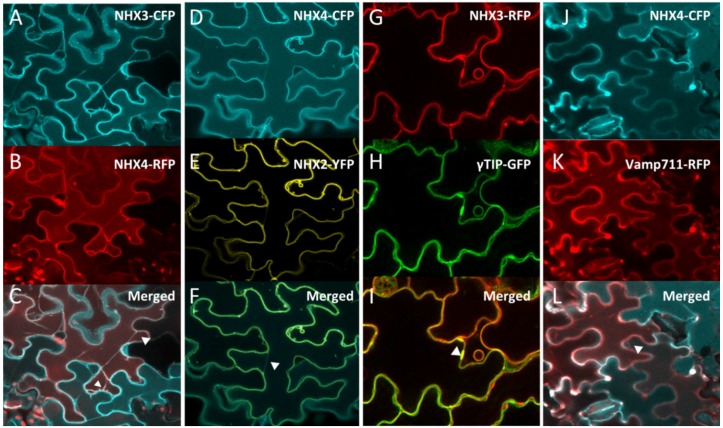
Intracellular localization of *NHX3* and *NHX4*. Transient co-expression, in *Arabidopsis* cotyledon epidermal cells, of *35S::NHX3-CFP* (**A**) with *35S::NHX4-RFP* (**B**) colocalized as shown in the merge fluorescent panel (**C**). Transient expression of *35S::NHX4-CFP* (**D**) in stable *35S::NHX2-YFP* background (**E**) also colocalized as shown in the merged image (**F**). Transient expression of *35S::NHX3-RFP* (**G**) in stable expressing γ*TIP::*γ*TIP-GFP* (**H**) colocalized as shown in the merged panel (**I**). Transient expression of *35S::NHX4-CFP* (**J**) in the stable expressing *Ub10::VAMP711-RFP* (**K**) colocalized as shown in the merged image (**L**). Arrowheads in (C,F,I,L) point to tonoplast invaginations and transvacuolar cytoplasmic strands.

To determine the subcellular localization of NHX3 and NHX4, we generated constitutively expressed translational fusion proteins of both NHX3 and NHX4 with CFP and RFP, respectively. Transient co-expression of NHX3-CFP and NHX4-RFP in *Arabidopsis* cotyledons displayed overlapping expression at the periphery of the cell, as well as at transvacuolar strands (Figure 1A,C). Because NHX1 and NHX2 were shown previously to localize at the tonoplast [3], we transiently expressed NHX4-CFP in plants stably expressing NHX2-YFP and observed an overlapping expression pattern between NHX2 and NHX4 that was characteristic of a vacuolar signal, including trans-vacuolar strands and vacuolar bulbs (arrowheads in Figure 1D–F). We then expressed NHX3-RFP and NHX4-CFP in aquaporin γ-TIP [14] and the R-SNARE VAMP711 [15] expressing lines, which were previously shown to colocalize with NHX1 [3]. In cotyledon epidermal cells, an overlapping expression between NHX3-RFP and γ-TIP-GFP, including in vacuolar bulbs that are characteristic of γ-TIP, was seen (Figure 1G–I). Overlapping expression was also seen with NHX4-CFP and VAMP711-RFP (Figure 1J–L). Taken together, these results indicated that both NHX3 and NHX4 localize to the tonoplast, as suggested by other heterologous expression studies [5,7] and sequence similarity to NHX1 and NHX2 [1].

### 2.2. Growth Phenotypes of nhx1nhx2nhx3nhx4 and Responses to Na^+^, K^+^

**Figure 2:**
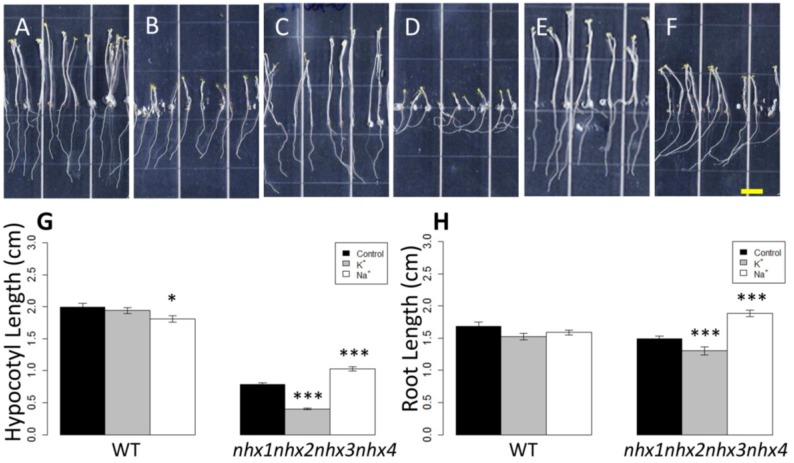
Etiolation response of the knockout *nhx1nhx2nhx3nhx4*. Etiolated wild-type (**A**,**C**,**E**) and *nhx1nhx2nhx3nhx4* (**B**,**D**,**F**) seedlings grown for 11 days in the dark on either 30 mM K^+^ (**C**,**D**) or 30 mM Na^+^ media (**E**,**F**). No significant difference in hypocotyl or root growth was noted in the wild-type; however, in *nhx1nhx2nhx3nhx4*, root and hypocotyl growth was significantly inhibited in seedlings grown in 30 mM K^+^ and increased in those grown in 30 mM Na^+^, as compared to control media. Quantification of hypocotyl (**G**) and root (**H**) growth normalized relative to growth on control media (1 mM Na^+^ and 1 mM K^+^). *nhx1nhx2nhx3nhx4* displayed left-handed root skewing when grown on media containing 30 mM K^+^ (**D**), but not 1 mM K^+^ (**B**). Values represent the mean ± SE (*n* = 29). * *p* ≤ 0.05, *** *p* ≤ 0.001; *p*-values reflect significant differences between control and either K^+^ or Na^+^ treatments within each genotype. The yellow bar is 0.5 cm.

**Figure A2:**
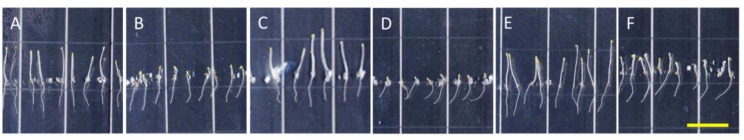
Etiolation response of four-day-old *nhx1nhx2nhx3nhx4*. WT (**A**,**C**,**E**) and *nhx1nhx2nhx3nhx4* (**B**,**D**,**F**) grown on 1 mM K^+^, 1 mM Na^+^ (**A**,**B**), 30 mM K^+^ (**C**,**D**) or 30 mM Na^+^ (**E**,**F**). Root growth of *nhx1nhx2nhx3nhx4* does not differ between treatments, but hypocotyls are shorter on 30 mM K^+^ (**D**) than 1 mM K^+^, 1 mM Na^+^ (**B**) or 30 mM Na^+^ (**F**). Growth of both roots and hypocotyls in WT is unaffected across treatments. The yellow bar is 1 cm.

We used the etiolation response of dark-grown seedlings to examine the contribution of *NHX1*, *NHX2*, *NHX3* and *NHX4* to cell expansion and seedling growth. Previously, we reported that etiolated *nhx1nhx2* double knockout seedlings had profound hypocotyl and root elongation phenotypes [3]. Etiolated *nhx1nhx2nhx3nhx4* seedlings displayed significantly reduced growth of both roots and hypocotyls (Figure 2). In control media, hypocotyl lengths of *nhx1nhx2nhx3nhx4* were less than 50% the length of WT after 11 days of etiolated growth (Figure 2M). The hypocotyl elongation response of *nhx1nhx2nhx3nhx4* was highly responsive to both added Na^+^ and K^+^. For example, hypocotyls of *nhx1nhx2nhx3nhx4* grown in 30 mM NaCl were 31% longer than those of seedlings grown on control media (1 mM NaCl, 1 mM KCl), but hypocotyls of *nhx1nhx2nhx3nhx4* seedlings grown in 30 mM KCl were 49% shorter (Figure 2D,H,K,L) than those grown in control media. By contrast, little change in hypocotyl length was observed in the WT seedlings grown on either 30 mM NaCl or KCl (Figure 2B,F,J,L). Root growth was also markedly different between *nhx1nhx2nhx3nhx4* and WT vertically-grown seedlings, but only in response to either K^+^ or Na^+^. Similar to the K^+^ and Na^+^-dependent growth response of hypocotyls, *nhx1nhx2nhx3nhx4* roots were also significantly shorter (26%) under K^+^ and longer (13%) under Na^+^ as compared to *nhx1nhx2nhx3nhx4* grown in control media. In addition, the *nhx1nhx2nhx3nhx4* seedlings began to display prominent left-handed skewing (as viewed through the agar, following the convention assigned by Rutherford and Masson [16]) after four days of growth on media supplemented with 30 mM K^+^ (Figure A2). Seedlings grown for another seven days on the same media displayed complete curling of roots (Figure 2H). By contrast, WT root growth remained unchanged under 30 mM K^+^ even after 11 days.

Previously, we reported that etiolated *nhx1nhx2* plants also displayed reduced hypocotyl growth, that was 70% the length of WT hypocotyls on media containing 1 mM K^+^ and 50% of WT on 30 mM K^+^ [3], while in etiolated *nhx1nhx2nhx3nhx4* seedlings, the average hypocotyl length was 40% and 20% of WT on 1 and 30 mM K^+^, respectively. A comparison of root growth responses between *nhx1nhx2nhx3nhx4* and *nhx1nhx2* to high K^+^ [3] indicates that root curling is also more pronounced and root growth more sensitive to high K^+^, similar to that observed in hypocotyls. The dramatic differences in root and hypocotyl elongation between *nhx1nhx2* and *nhx1nhx2nhx3nhx4* under the higher K^+^ suggest that NHX3 and NHX4 have additional roles in K^+^ homeostasis and cell expansion.

**Figure A3:**
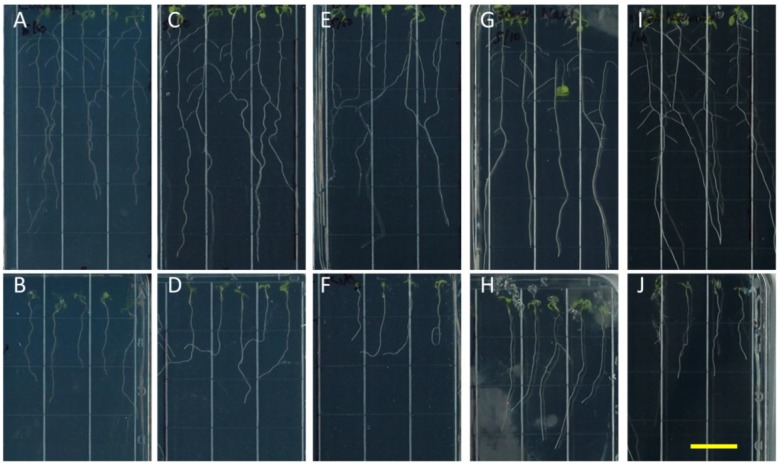
*nhx1nhx2nhx3nhx4* root tip curling is K^+^ specific. Six-days-old WT (**A**,**C**,**E**,**G**) and *nhx1nhx2nhx3nhx4* (**B**,**D**,**F**,**H**). Both WT and *nhx1nhx2nhx3nhx4* exhibit straight root growth on media containing 1 mM KCl (**A**,**B**). When grown on media supplemented with either 30 mM KCl (**C**,**D**) or 30 mM KNO_3_ (**E**,**F**), WT roots grow normally, whereas roots of *nhx1nhx2nhx3nhx4* exhibit a left-handed curling. Neither 30 mM NaCl (**G**,**H**) nor 60 mM mannitol (**I**,**J**) induced root curling, although the addition of NaCl produced longer root growth in *nhx1nhx2nhx3nhx4* (**H**) compared with other treatments. The yellow bar is 1 cm.

To determine if the root curling observed in *nhx1nhx2nhx3nhx4* roots was specific to K^+^, we compared the root growth of *nhx1nhx2nhx3nhx4* on media supplemented with KCl, KNO_3_, NaCl or mannitol. Mannitol was included in order to determine whether the root curling response of *nhx1nhx2nhx3nhx4* under high K^+^ was due to an osmotic effect; thus, 60 mM mannitol (60 mOsm, equivalent to the osmolarity of the added salt) was used. We found that *nhx1nhx2nhx3nhx4* root growth on mannitol was not different from the control (Figure A3J,B), and therefore not likely due to an osmotic effect. As shown in Figure A3, both 30 mM KCl and 30 mM KNO_3_ supplemented media resulted in similar left-handed root skewing and curling of the root tip that was not seen when *nhx1nhx2nhx3nhx4* was grown on mannitol, suggesting that the response of *nhx1nhx2nhx3nhx4* was specific to K^+^. Collectively, experiments comparing *nhx1nhx2nhx3nhx4* seedling growth (*i.e.*, Figure 2 and Figure A3) indicate that the knockouts’ root curling response was caused by aberrant K^+^ homeostasis.

Previous work has provided a strong link between ion stress and directional root growth. For example, *sos1*(*nhx7*) null mutants displayed a loss of normal gravitropic root growth when grown vertically on media supplemented with high (50–150 mM) Na^+^, with young roots exhibiting skewing and eventual upward directed growth [17]. Moreover, roots of both WT and *sos1*(*nhx7*) seedlings grown on high Na^+^ showed delayed gravitropic responses, thereby suggesting that aberrant intracellular ion homeostasis can specifically affect directional root growth. The *sos1* skewing phenotype is remarkably similar to *nhx1nhx2nhx3nhx4* grown on K^+^ supplemented media, and although effects of high cytosolic Na^+^ have been suggested, the effects of K^+^ on root skewing remain unclear. Furthermore, *sos1*(*nhx7*) mutants were more sensitive to microtubule reorganization, with whole mount immunostaining experiments in elongating root epidermal cells demonstrating that *sos1* (*nhx7*) in the presence of 50 mM Na^+^ had helically-orientated microtubules, while WT plants maintained the normal transverse array [12]. Although microtubule remodeling has been shown to be induced by high external Na^+^, reports of K^+^-induced microtubule reorganization are sparse [12,13,18]. In KCl (350 mM)-treated *Zea mays*, a reorganization of the cortical microtubule array from the transverse to oblique orientation in root cells of the maturation zone occurred within 3 min after application. By contrast, treatment with sorbitol did not result in the reorganization of microtubules [19]. These results suggested that cortical microtubule organization in roots was affected by high concentrations of Na^+^ and K^+^. Given the phenotypes of *nhx1nhx2nhx3nhx4*, the knockout represents a useful tool to study the role of K^+^ in cytoskeletal organization. The positive effect of Na^+^ and the negative effect on K^+^ on *nhx1nhx2nhx3nhx4* growth is well supported by similar results obtained in *nhx1nhx2* in which Na^+^ was proposed to substitute for the lack of K^+^ accumulation in vacuoles, providing the osmoticum needed to generate the turgor for cell expansion [3,10]. The sensitivity of both *nhx1nhx2* and *nhx1nhx2nhx3nhx4* to added K^+^, as well as the aberrantly high cytosolic K^+^ concentrations of *nhx1nhx2*, together with previously reported effects of Na^+^ on cytoskeleton disassembly and reorganization, suggests that aberrant K^+^ homeostasis in the *nhx1nhx2nhx3nhx4* might affect the organization of the cytoskeleton.

### 2.3. nhx1nhx2nhx3nhx4 Lacks Accumulation of K^+^ in the Vacuole

Previously, we quantified the concentration of K^+^ in vacuoles of the knockout *nhx1nhx2* using the K^+^ binding dye, PBFI [3]. Using the same approach, we quantified the vacuolar [K^+^] of *nhx1nhx2nhx3nhx4* in root tip cells of the elongation zone, where K^+^-induced root curling was observed (Figure 2D). In *nhx1nhx2nhx3nhx4*, vacuolar K^+^ was ~19 mM (±7 mM) and significantly lower than WT, which was 77 mM (±13 mM). These values are similar to those obtained in *nhx1nhx2*, suggesting that the lack of *NHX3* and *NHX4* in addition to *NHX1* and *NHX2* did not significantly alter root tip cell vacuolar K^+^. In the same *nhx1nhx2* knockout, the cytosolic K^+^ was also reported to be aberrantly high compared to WT [9]. Therefore, given that the electrochemical potential of K^+^ favors uptake into the cell [1,3], it is possible that in *nhx1nhx2nhx3nhx4*, the lack of K^+^ accumulation into the vacuole might lead to an aberrantly high K^+^ accumulation in the cytosol.

### 2.4. Cells of the Root Transition Zone Lack Expansion in nhx1nhx2nhx3nhx4

**Figure 3:**
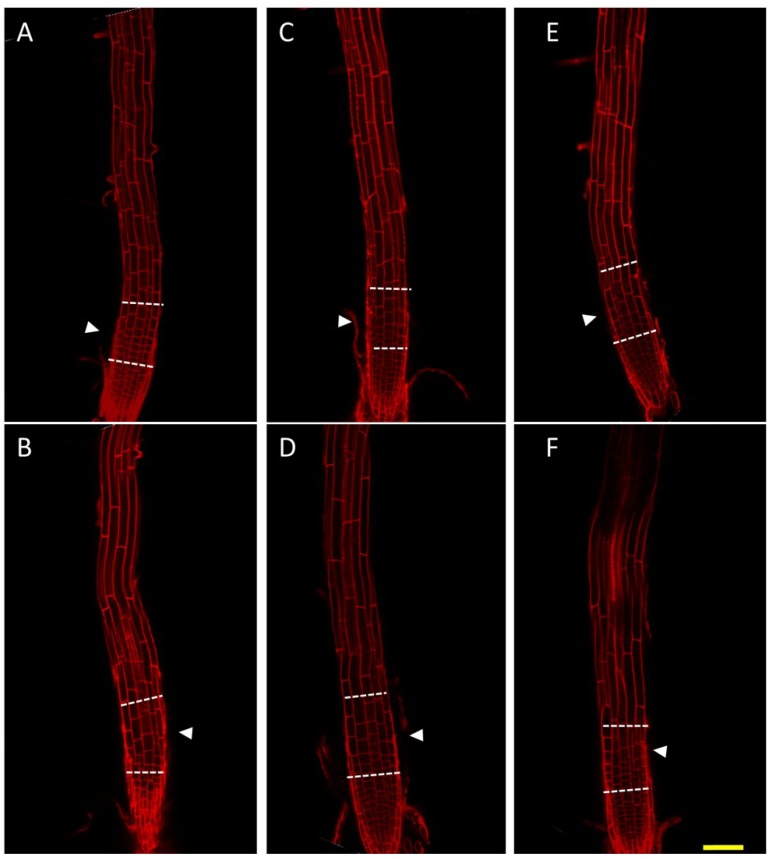
Root cell expansion in the *nhx1nhx2nhx3nhx4* root transition zone is reduced. WT (**A**,**C**,**E**) or *nhx1nhx2nhx3nhx4* (**B**,**D**,**F**) six-day-old dark-grown seedlings were stained with 10 µg·mL^−1^ propidium iodide. Seedlings were grown on control media (1 mM K^+^, 1 mM Na^+^) (**A**,**B**) or on media supplemented with 30 mM NaCl (**C**,**D**) or 30 mM KCl (**E**,**F**). The transition zone is bounded by the dashed lines. Arrowheads point to cells that show differences in cell size and are discussed in the Results. Bar is 50 μm.

To investigate the significance of NHX1 to NHX4-mediated Na^+^ and K^+^ transport on cell expansion and root skewing, we stained roots of *nhx1nhx2nhx3nhx4* seedlings with propidium iodide and visualized cell size. Compared with WT, cells in the *nhx1nhx2nhx3nhx4* root transition zone appeared to be much shorter when grown on 30 mM KCl-supplemented media (Figure 3E,F). Cell size was similar between WT and *nhx1nhx2nhx3nhx4* in the apical meristem, but epidermal cells in the transition zone were markedly smaller in *nhx1nhx2nhx3nhx4*. Whereas in the WT, a gradual increase in cell size from the meristem to the elongation zone (Figure 3E, arrows) was apparent, the length of epidermal cells in the transition zone of *nhx1nhx2nhx3nhx4* was aberrantly small, and as a consequence, the transition zone appeared “compressed” (Figure 3F, white arrows). This phenotype was not evident in *nhx1nhx2nhx3nhx4* grown on 30 mM NaCl-supplemented media, where no difference in cell size or shape was observed between WT and *nhx1nhx2nhx3nhx4* in any root zones (Figure 3C,D).

### 2.5. K^+^ Dependent Root Skewing Depends on the Dynamic Organization of the Cytoskeleton

**Figure 4:**
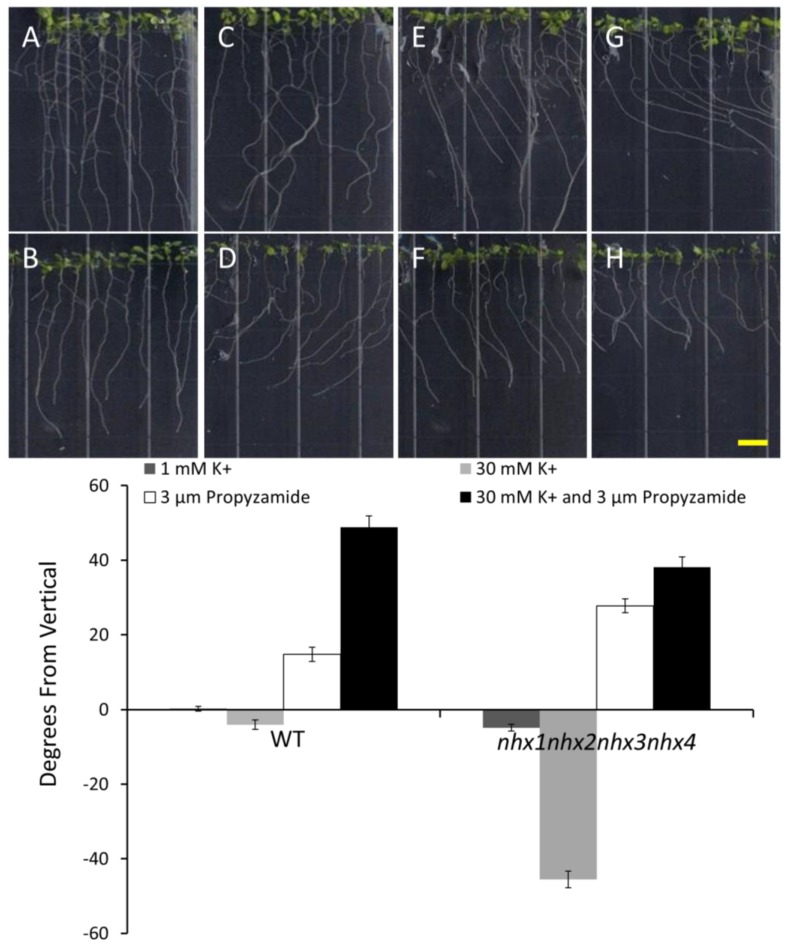
Propyzamide alters potassium-induced root skewing in the *nhx1nhx2nhx3nhx4* knockout. Wild-type (**A**,**C**,**E**,**G**) and *nhx1nhx2nhx3nhx4* (**B**,**D**,**F**,**H**) seedlings were germinated on minimal media and transferred to plates with either 30 mM K^+^ (**C**,**D**), 3 µM propyzamide (**E**,**F**) or both 30 mM K^+^ and propyzamide (**G**,**H**) when four day-old and grown for an additional seven days. Root skewing is quantified as the angle of root growth in degrees from vertical (**I**). Values represent the mean ± SE (*n* = 28). The bar is 0.5 cm.

In order to investigate whether the root growth response of *nhx1nhx2nhx3nhx4* to elevated K^+^ concentrations was associated with dynamic changes in the organization of the cytoskeleton, we tested the effect of microtubule perturbing drugs on root morphology and directional root growth in etiolated *nhx1nhx2nhx3nhx4* seedlings as above. Propyzamide is a microtubule destabilizing drug that is known to induce right-handed root skewing (when viewed through the agar) without inhibiting overall growth and root elongation [12,20,21]. When applied at 3 μM, anisotropic root growth of wild-type *Arabidopsis* is moderately impaired without causing swelling of epidermal roots cells [22]. We measured root growth angles for both WT and *nhx1nhx2nhx3nhx4* vertically-grown seedlings on minimal media (containing 1 mM KCl) and an identical media supplemented with an additional 30 mM KCl. As shown in Figure 4, both the WT and *nhx1nhx2nhx3nhx4* displayed the characteristic right-handed skewing (Figure 4E,F) when grown in the presence of 3 µM propyzamide, but only *nhx1nhx2nhx3nhx4* displayed left-handed skewing in response to 30 mM K^+^ (Figure 4D). When both elevated K^+^ and 3 µM propyzamide were included in the media, roots from WT seedlings continued to skew towards the right, unlike the roots from *nhx1nhx2nhx3nhx4*, which displayed less pronounced skewing (Figure 4G,H).

Wang and colleagues demonstrated that WT and *sos1*(*nhx7*) seedlings exhibited left-handed root skewing when grown vertically on NaCl-enriched MS media, similar to the root skewing of *nhx1nhx2nhx3nhx4* in the presence of high KCl. Their results indicated that 50 mM Na^+^ was sufficient to initiate root skewing that eventually resulting in strong root curling and the formation of root “loops”. Interestingly, Na^+^-dependent root skewing was exacerbated in *sos1*(*nhx7*) mutants, which displayed more prominent root skewing than the WT at lower Na^+^ treatments, suggesting that cytosolic ion accumulation played an important role in directional root growth. NaCl-induced salt stress resulted in seedling death, which occurred more frequently in *sos1*(*nhx7*) than WT seedlings, but was partially prevented by propyzamide treatment, suggesting that microtubule reorganization was required for salt tolerance [18].

**Figure 5:**
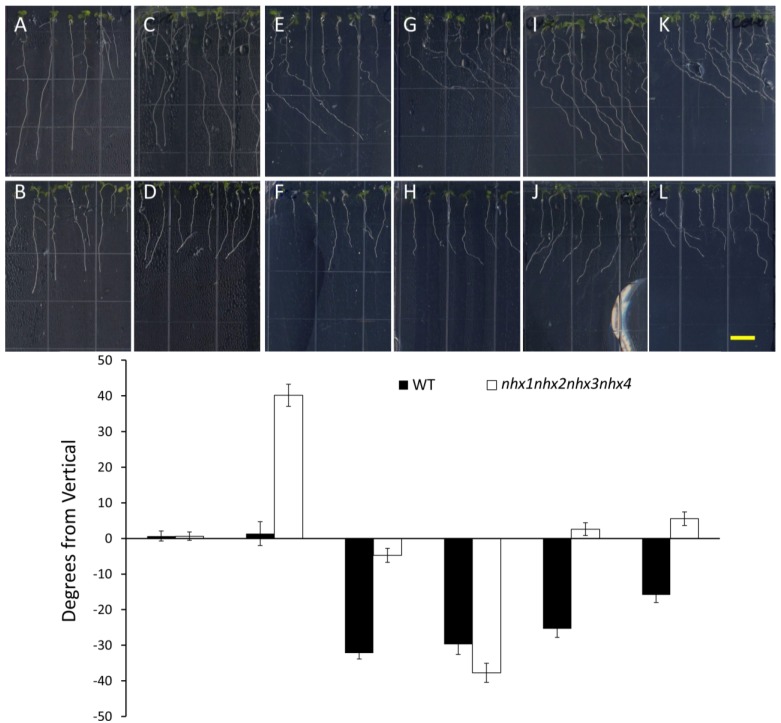
Taxol reverses directional root growth in *nhx1nhx2nhx3nhx4* grown on potassium-supplemented media. Wild-type (**top**) and *nhx1nhx2nhx3nhx4* (**bottom**) seedlings were germinated on minimal media containing 1 mM K^+^ for three days and transferred to plates containing two concentrations of Taxol and K^+^ (indicated below) and grown for an additional four days. (**A**,**B**) 1 mM K^+^; (**C**,**D**) 30 mM K^+^; (**E**,**F**) 0.5 µM Taxol and 1 mM K^+^; (**G**,**H**) 1 µM Taxol and 1 mM K^+^; (**I**,**J**) 0.5 µM Taxol and 30 mM K^+^; (**K**,**L**) 1 µM Taxol and 30 mM K^+^. (**M**) The angle of root growth was quantified as the degrees from vertical; values represent the mean ± SE (*n* = 24). The bar is 0.5 cm.

We also tested the effect of Taxol, a microtubule stabilizing drug, on K^+^-induced root skewing in *nhx1nhx2nhx3nhx4* plants*.* A characteristic effect of Taxol on roots is a right-handed skewing response that is dose dependent [23]. We observed that 0.5 µM Taxol was sufficient to induce right-handed root skewing in the WT (Figure 5E), but the same concentration did not significantly change the root growth direction of *nhx1nhx2nhx3nhx4* plants (Figure 5F). It is unlikely that the lack of *nhx1nhx2nhx3nhx4* root skewing observed in the 0.5 µM Taxol treatment was caused by a lack of overall root growth, because *nhx1nhx2nhx3nhx4* roots did not vary significantly in length between 0.5 µM or 1.0 µM Taxol treatments. Indeed, the addition of 30 mM KCl was sufficient to induce left-handed skewing in *nhx1nhx2nhx3nhx4*, despite the presence of 0.5 µM Taxol (Figure 5J). In contrast, the added KCl had no effect on WT root growth, with seedlings continuing to skew to the right (Figure 5I). We observed that a threshold of 1.0 µM Taxol was sufficient to overcome the K^+^-induced left-handed skewing in *nhx1nhx2nhx3nhx4* plants and to cause right-handed skewing, as shown (Figure 5L). Previously, it was shown that 0.5 μM Taxol counters the effect of Na^+^-induced root skewing, which is dose dependent [18]; a result that is strikingly similar to what we observed in Taxol- and K^+^-treated *nhx1nhx2nhx3nhx4* (Figure 5).

To better understand the possible effect of high K^+^ on cytoskeletal organization, we used a whole mount immunostaining approach to visualize the organization of cortical microtubules in cells of the root elongation zone, where pronounced skewing and curling occurred.

**Figure 6:**
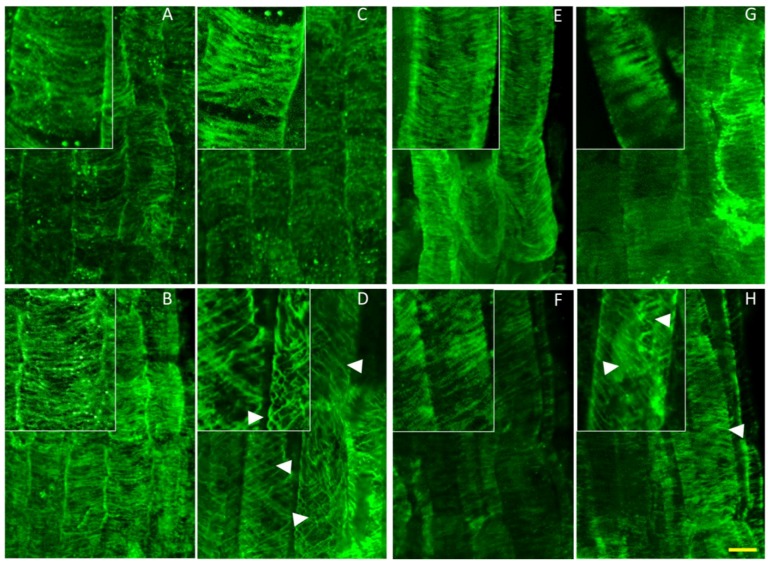
Potassium induces helical reorganization of cortical microtubules of *nhx1nhx2nhx3nhx4* epidermal cells in the root elongation zone. Immunolabeled microtubules in WT (top) and *nhx1nhx2nhx3nhx4* (bottom). Seedlings were germinated on minimal media containing 1 mM K^+^ for three days, then transferred to plates with two concentrations of Taxol and grown for an additional five days before fixation. Normal microtubule orientation is observed in WT (**A**) and *nhx1nhx2nhx3nhx4* (**B**) when grown on media containing 1 mM K^+^. WT seedlings grown on 30 mM K^+^ displayed normally-orientated microtubules (**C**), whereas 30 mM K^+^ induced a helical orientation of the cortical microtubules in *nhx1nhx2nhx3nhx4* (**D**). In the presence of 1 mM K^+^ and 0.5 µM Taxol, both WT (**E**) and *nhx1nhx2nhx3nhx4* (**F**) display characteristic bundling of cortical microtubules. Whereas 30 mM K^+^ had no effect on microtubule orientation in WT grown with 0.5 µM Taxol (**G**), *nhx1nhx2nhx3nhx4* seedlings grown with 30 mM K^+^ and 0.5 µM Taxol (**H**) exhibited a helical microtubule organization similar to that seen when grown with just 30 mM K^+^. Arrows point to helically-oriented microtubules. The bar is 10 μM. The upper left insets are enlarged 50% to highlight microtubule orientation.

As depicted in Figure 6, no differences were observed between the microtubule organization of roots from *nhx1nhx2nhx3nhx4* and the WT when grown on media containing 1 mM K^+^ (Figure 6A,B). By contrast, *nhx1nhx2nhx3nhx4* seedlings grown on media containing 30 mM K^+^ displayed a helical orientation of the cortical microtubule array in elongating *nhx1nhx2nhx3nhx4* root cells (Figure 6D) that was observed only when roots exhibited left-handed skewing. Both WT and *nhx1nhx2nhx3nhx4* displayed the characteristic Taxol-induced “bundling” of microtubules [24,25] with a highly parallel microtubule organization (Figure 6E,F). However, when seedlings were grown on media with both 0.5 µM Taxol and 30 mM KCl, the *nhx1nhx2nhx3nhx4* microtubules displayed a similar spiraling pattern that was seen when grown on 30 mM KCl plates (Figure 6H,D). Higher K^+^ had no observable effect on microtubule organization in the WT, even in the presence of Taxol (Figure 6C,G).

In particular, cortical microtubules depolymerize and reorganize in a Ca^2+^-dependent manner under salt stress; preventing this response with microtubule stabilizing drugs, such as Taxol, results in seedling death [12,13], suggesting that cytoskeletal reorganization of root cells is important during salt stress. In addition, mutations of the Na^+^/H^+^ antiporter SOS1 and associated SOS2 kinase were shown to suppress the cortical microtubule disruptions and subsequent helical growth associated with the *Arabidopsis spiral1* mutants. This study also demonstrated that even in the absence of salt stress, loss of function SOS mutants resulted in altered cortical microtubule arrays when seedlings were treated with the same dose of propyzamide as used in the present study [12].

## 3. Experimental Section

### 3.1. Plant Materials and Growth Conditions 

T-DNA insertions lines for *NHX1* (At5g27150), *NHX2* (At3g05030), *NHX3* (At5g55470) and *NHX4* (At3g06370) were obtained and were *nhx1-1* [SALK_034001], *nhx2-1* [SALK_036114], *nhx3-1* [WISC_345-348F19] and *nhx4-1* [SALK_112901] [26]. All single knockouts were backcrossed twice prior to crosses for generating higher order knockouts. Subsequent crosses generated double knockouts and two triple knockouts, which were then crossed to generate a tetra *nhx* knockout. RT-PCR confirmed no DNA amplification with allele-specific primers from leaf-derived *nhx1nhx2nhx3nhx4* cDNA. Positions of T-DNA insertion sites and RT-PCR results are shown in Figure A1.

*Arabidopsis thaliana* (ecotype Columbia [Col-0]) and *nhx1nhx2nhx3nhx4* seedlings were grown at 22 °C under diurnal light conditions (8 h light and 16 h dark), unless otherwise specified. Seeds were germinated on vertically-positioned plates containing a modified Murashige and Skoog media [27] (0.5% sucrose, 1 mM Na^+^, 1 mM K^+^, 0.8% phytagel, pH 5.7) and supplemented as specified for each experiment. For experiments testing stress responses, this media was considered as a control and was modified by the addition of 30 mM NaCl or KCl or 60 mM mannitol. For drug treatment assays, media was supplemented with 3 µM propyzamide (Sigma cat.#45645) or either 0.5 µM or 1 µM Taxol (Sigma cat.#T7191).

### 3.2. Cloning and Expression of Translational Fusion Proteins

*NHX3* and *NHX4* translational fusions were constructed using the Gateway cloning system (Invitrogen, Carlsbad, CA, USA). cDNAs without stop codons were cloned into pDONR207 to generate entry vectors (pDONR207-NHX2, pDONR207-NHX3, pDONR207-NHX4) and recombined into pEarley-Gate101 for YFP fusion and pEarleyGate102 for CFP fusion [28]. For the 35S-NHX4-RFP construct, pDONR207-NHX4 was recombined with pH7RWG2 [29]. All constructs were introduced to *A. tumefasciens* strain GV3101 for both stable and transient transformation of Col-0, VAMP711-RFP [30] and γ-TIP-GFP.

### 3.3. Transient Transformation of Arabidopsis Seedlings

Transient transformation of *Arabidopsis* was adopted from Li, *et al.* [31] using 4–5-day-old seedlings grown in a sterile 24-well plate. Seedlings were co-cultivated with *Agrobacterium tumefasciens* (strain GV3101) for 18–24 h before observation. Co-transformation using two constructs (Figure 1I,L) was accomplished by co-cultivating seedlings with two bacterial cultures, each diluted to one half of the final optical density used when transforming a single construct (OD 0.5).

### 3.4. Hypocotyl and Root Length Measurements

**Figure A4:**
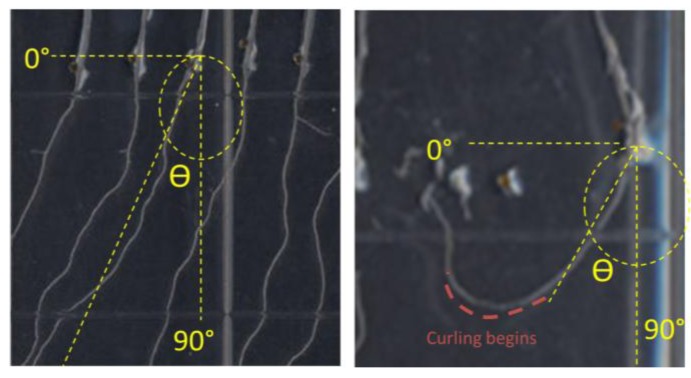
Root skewing measurement methodology. The etched grid on the back of square petri plates served as a reference point for assigning the vertical and horizontal vectors. Growth vectors were measured by drawing a line along the general axis of root growth. For curling roots, the growth vector was determined before the point at which the root begins to curl.

Seedlings grown on agar plates were scanned on a flatbed scanner as color TIFF files with 300-dpi resolution. Root and hypocotyl lengths were quantified using the ImageJ [32] plugin SmartRoot [33]. The angles of root growth were measured with the same software suite using gridlines etched on the rear of the petri dishes as horizontal and vertical reference points (see Figure A4).

### 3.5. Quantification of Vacuolar K^+^

Vacuolar K^+^ was quantified using the K^+^-specific dye PBFI-AM, as described previously [3].

### 3.6. Propidium Iodide Staining

Dark-grown, six-day-old WT and *nhx1nhx2nhx3nhx4* seedlings were grown as described above. Seedlings were stained with a 10-µg·mL^−1^ solution of propidium iodide (Sigma cat.#81845).

### 3.7. Whole Mount Immunostaining of Arabidopsis Seedlings

Whole mount immunostaining of root cortical microtubules was modified from the protocol of Hauser *et al.* [34]. Dark-grown seedlings grown for five days after germination were fixed on agar plates in a solution of 4% p-formaldehyde, 5% DMSO, 0.025% glutaraldehyde in 1× microtubule stabilizing buffer (MTSB; 50 mM PIPES, 5 mM EGTA, 5 mM MGSO_4_, pH 7) vacuum infiltrated at room temperature for 1 h. Seedlings were then washed three times in wash buffer (MTSB with 5% DMSO) three times for ten minutes each, and the solution was exchanged for a cell wall digestion buffer (MTSB with 1% cellulase, 0.1% pectolyase) and incubated at room temperature for 30 min. Following digestion, three washes were performed with MTSB for five minutes each, and the seedlings were incubated for ten minutes in a solution of MTSB with 1 mg/mL NaBH_4_ for ten minutes. After three more washes with MTSB for five minutes, each seedling was incubated for ten minutes in a solution of MTSB containing 10 mM glycine and then washed once more with MTSB three times for a total of 30 min. Severed roots were transferred to microscope slides (Thermo-Scientific Superfrost plus, cat.#4951PLUS-001) and incubated in a membrane permeabilization solution (MTSB with 1% Triton X-100) at room temperature for 15 min and then in methanol at −20 °C for 20 min.

Immunostaining was carried out by an incubation with DM1A primary antibody (Sigma cat.#T9026) diluted 1:400 in a blocking solution (PBS containing 3% BSA, 0.05% Tween-20, and 0.1% thimerosal) for one hour at 37 °C. After washing, roots were incubated with FITC goat anti-mouse (Sigma cat. #F027) for one hour at 37 °C.

### 3.8. Confocal Microscopy

Fluorescence microscopy was performed using a confocal laser scanning microscope (Zeiss LSM 710) equipped with a 40× water immersion objective. The excitation wavelength was 488 nm, with emission wavelengths of 500–535 nm for GFP or FITC, 525–615 nm for YFP/Venus, 450–500 nm for CFP and 565–605 nm for RFP. For imaging of GFP/RFP and CFP/YFP double reporter lines, sequential scanning on multiple channels was used to avoid crosstalk between fluorescence emission spectra.

## 4. Conclusions

We previously reported that NHX1 and NHX2 colocalize to the vacuole and are essential for vacuolar K^+^ accumulation and the regulation of pH [3]. Here, we demonstrate that both NHX3 and NHX4 are also localized to the tonoplast and that vacuolar cation/H^+^ exchange by all four vacuolar NHXs are essential for normal growth and development. Compared to both WT and the *nhx1nhx2* double knockout, the quadruple knockout *nhx1nhx2nhx3nhx4* has significantly reduced vacuolar K^+^ and growth, as well as high sensitivity to added K^+^. *nhx1nhx2nhx3nhx4* also exhibited pronounced root skewing and curling responses not observed under equimolar Na^+^ that were correlated with an increase in cytoskeletal dynamics and a reduction in the size of the root cells of the transition zone.
